# COVID-somnia: anxiety, insomnia, and poor sleep among second-line healthcare workers during COVID-19 pandemic

**DOI:** 10.1186/s43168-022-00143-9

**Published:** 2022-08-11

**Authors:** Torki Al-Otaibi, Ahmad Abbas, Ayman Maher Nagib, Osama Ashry Gheith, Prasad Nair, Mahmoud M. Farid, Mohammad A. S. Albader

**Affiliations:** 1 The Nephrology Department, Hamed Al-Essa Organ Transplant Center, Ibn Sina Hospital, Sabah area Kuwait City, Kuwait; 2 Chest Department, Faculty of Medicine, Zagazig University, Zagazig, Egypt; 3 Department of Dialysis and Transplantation, Urology Nephrology Center, Mansoura University, Mansoura, Egypt; 4 Clinical Pathology, National Blood Transfusion Services, Cairo, Egypt; 5 Health Care Management, Technical Office, MOH, Kuwait City, Kuwait

**Keywords:** Anxiety, COVID-19, COVID-somnia, Insomnia, Sleep quality

## Abstract

**Background:**

Little information is available about the linkage between sleep affection and COVID-19. Preliminary reports and clinical observations focused on the appearance of related mental health issues, especially in healthcare workers (HCWs).

**Methods:**

A cross-sectional study is conducted on the COVID-19 second-line HCWs using an English online survey prepared via Google forms. The survey focused on sociodemographic and profession-related characteristics (age, sex, smoking, history of previous sleep disorders or medications affecting sleep, comorbidities specialty, years of experience, and number of hours worked per week) and COVID-19-associated risks (being on the second line of COVID-19 management, following updates and news about COVID-19, and getting an infection with COVID-19 or having a colleague/friend who was infected with or died of COVID-19). Assessment of anxiety, insomnia, and sleep quality was done using the relevant diagnostic scales.

**Results:**

This study included 162 second-line HCWs with a mean age of 34.36 ± 8.49 years. Although being in second lines, there was a high prevalence of anxiety (49.38%), insomnia (56.17%), and poor sleep quality (67.9%) during the pandemic. One condition was recently developed after the pandemic: insomnia in 6.6%, anxiety in 5.7%, and poor sleep in 16%. Two conditions were developed: insomnia and poor sleep in 21.7%, anxiety and poor sleep in 7.5%, and insomnia and anxiety in 10.4%. The three conditions were de novo experienced in 19.8%. A total of 22.4% of those who followed daily COVID-19 updates developed de novo combined anxiety, insomnia, and poor sleep. A total of 38.5% of participants that had been infected with COVID-19 developed de novo combined anxiety, insomnia, and poor sleep. A total of 50% of participants who had a colleague/friend who died with COVID-19 developed de novo combined anxiety, insomnia, and poor sleep.

**Conclusion:**

Although being in second lines, there was a high prevalence of anxiety, depression, and poor sleep concerning COVID-19-related factors.

## Introduction

The coronavirus disease (COVID-19) pandemic, caused by severe acute respiratory syndrome coronavirus 2 (SARS-CoV-2), was the hallmark of 2020. It has forced people to either stay at home or follow strict social distancing and telecommunication measures with a significant impact on the sleep schedule of a sizable population in terms of quality and quantity [1, 2].

During the current pandemic, sleep deterioration has been referred to as COVID-somnia. This global event, being a productive environment for the development of new sleep and mental health issues in those who had not experienced such events before, could have led to the release of sleep disorders, e.g., insomnia and restless legs syndrome that improved with the clearance of infection. As reported from early reports and surveys, frontline healthcare workers and infected persons are the groups of increased risk [3–7].

However, to the best of our knowledge, the second-line HCWs was not studied well during this pandemic. So, we conducted the current work to assess anxiety, insomnia, and poor sleep among behind-line health HCWs during the COVID-19 pandemic.

## Material and methods

### Study design

A cross-sectional study is conducted on the COVID-19 second-line HCWs working in the Ministry of Health, Kuwait, from August 2020 to April 2021.

### Sample size

As our study objective was to detect a significant change in frequency of anxiety, insomnia, or poor sleep due to the COVID-19 pandemic by using McNemar’s statistics, a pilot study was done on 20 subjects. We found that 0% of subjects had shifted from anxiety positive to anxiety negative versus 30% of subjects who had shifted from anxiety negative to anxiety positive, so at 80% power and 95% confidence interval (CI), the estimated minimum sample will be 24 pairs. We found that 0% of subjects had shifted from insomnia positive to insomnia negative versus 30% of subjects who had shifted from insomnia negative to insomnia positive, so at 80% power and 95% CI, the estimated minimum sample will be 24 pairs. We found that 0% of subjects had shifted from poor sleep positive to poor sleep negative versus 35% of subjects who had shifted from poor sleep negative to poor sleep positive, so at 80% power and 95% CI, the estimated minimum sample will be 20 pairs (MedCalc 13 for windows, MedCalc software bvba). After receiving all responses and exclusion of invalid ones, we draw our sample from the target population using simple random sampling.

### Exclusion criteria

Refusal to participate and respondents with incomplete or duplicate answers were excluded.

### Study definitions

- Frontline HCWs: The subset of essential workers is likely at highest risk for work-related exposure to SARS-CoV-2 because their work-related duties must be performed onsite and involve being nearby (< 6 ft) to the public or to coworkers with suspected or infected with COVID-19 such as pulmonologists, anesthesiologists, and intensivists.
- Second-line HCWs: Any HCW in specialties not in direct close contact with suspected or confirmed COVID-19 patients in their work without risk of work-related exposure to SARS-CoV-2 because of their work-related duties such as dermatologist, clinical pathologist, hematologist, surgeons, and nephrologists.

### Data collection

An English online survey was conducted via Google forms. Participants from different medical specialties were invited to complete this survey via social network platforms via an online web links. Google forms save each filled questionnaire in the principal investigator’s Google Drive. Respondents were assured of anonymity and confidentiality.

Consent to participate was the first question, and answering “yes” was mandatory. Ethical approval was obtained from the Research Ethics Committee, Ministry of Health, Kuwait (number of acceptance: 1464/2020). The study was conducted following the Declaration of Helsinki on human research.

We applied the survey two times: first, we asked the participants to provide answers about the pre-COVID-19 pandemic and, on the second round, to provide answers during the pandemic. The two occasions were applied 1 month apart to prevent the participants from being influenced by their prior answers.

The survey focused on the following:

- Sociodemographic and profession-related characteristics: age, sex, smoking, marital status, number of siblings, partner’s job, history of previous sleep disorders or medications affecting sleep, comorbidities specialty, current job title, years of experience, and number of hours worked per week.
- COVID-19-associated risks: being on the second line of COVID-19 management, following updates and news about COVID-19, and getting an infection with COVID-19 or having a colleague/friend who was infected with or died of COVID-19.
- Assessment of anxiety using the Generalized Anxiety Disorder-7 scale (GAD-7), Arabic version: it is calculated by choosing scores of 0, 1, 2, and 3 corresponding to the categories of “not at all,” “several days,” “more than half the days,” and “nearly every day,” respectively, and then adding the scores for the seven questions together. Scores of 5, 10, and 15 are the cutoff points for mild, moderate, and severe anxiety, respectively [5].
- Assessment of insomnia with the following diagnostic criteria: (1) difficulty falling asleep, staying asleep, or non-restorative sleep, (2) this difficulty is present despite adequate opportunity and circumstance to sleep, (3) this impairment in sleep is associated with daytime impairment or distress, and (4) this sleep difficulty occurs at least three times per week and has been a problem for at least one month [6].
- Sleep quality was assessed using the Pittsburgh Sleep Quality Index. A total score of more than 5 is a diagnostic of poor sleep [7].

### Statistical analysis

All data were collected, tabulated, and statistically analyzed using SPSS 22.0 for windows (IBM Inc., Chicago, IL, USA). Continuous variables were expressed as the mean ± SD and median (range), and the categorical variables were expressed as a number (percentage). Percent of categorical variables was compared using Pearson’s chi-square test or Fisher’s exact test when was appropriate. Comparison between paired categorical variables was made by McNemar’s test. All tests were two sided. A *p*-value < 0.05 was considered significant.

## Results

This study included 162 second-line healthcare workers with a mean age of 34.36 ± 8.49 years, 38.27% were males, 12.35% were current smokers, 24.7% had a positive comorbid profile, and 39.51% married to a healthcare worker. Regarding the profession-related characteristics, 46.9% were registrars, 40.1% were senior registrars or specialists, and 13% were consultants. More than 50% had up to 5 years of experience, and 87.65% worked ≤ 48 h weekly (Table 1).

**Table 1:** Sociodemographic characters of the participants

Demographic factors	(*n* = 162)	
**Sociodemographic**	**Mean/*n***	**SD/%**
Age	34.36	8.49
**Age group**		
< 35 years	86	53.1
≥ 35 years	76	46.9
**Sex (male)**	62	38.27
**Current smoker (yes)**	20	12.35
**Comorbidity (yes)**	40	24.7
**Partner’s job (HCW)**	64	39.51
**Current job titles**		
Registrar	76	46.9
Senior registrar/specialist	65	40.1
Consultant	21	13
**Years of experience**		
1–5 years	84	51.85
6–10 years	16	9.88
> 10 years	62	38.27
**Number of working hours weekly**		
≤ 48 h	142	87.65
> 48 h	20	12.35

COVID-19-related factors that may affect the study participants were shown in Table 2, in which 79.01% of the participants were following updates about daily COVID-19 infections and deaths. Of them, 11.72% had been infected with COVID-19, and 64.2% and 6.79% knew a colleague/family member infected with or died from COVID-19, respectively.

**Table 2:** COVID-19-related factors among participants

Factors	(*n* = 162)	
	**No**	**%**
Following updates about COVID-19	128	79.01
I got infected with COVID-19	19	11.72
A family member or colleague infected with COVID-19	104	64.2
A family member or colleague who died from COVID-19	11	6.79

In the current work, the frequency of anxiety, insomnia, and poor sleep among studied HCWs before the COVID-19 pandemic was 11.7%, 15.4%, and 17.9%, respectively, as shown in Table 3. Although being in behind lines, there was a high prevalence of anxiety (49.38%), insomnia (56.17%), and poor sleep quality (67.9%) during the COVID-19 era, as shown in Table 4.

**Table 3:** Frequency of anxiety, insomnia, and poor sleep quality among participants before COVID-19 pandemic

Mental health problem	**Total (*n* = 162)**	
	No	%
Anxiety	19	11.7
Insomnia	25	15.4
Poor sleep	29	17.9

**Table 4:** Frequency of anxiety, insomnia, and poor sleep quality among participants during the COVID-19 pandemic

Variables	Total (*n* = 162)	
	**No**	**%**
Anxiety	80	49.38
Insomnia	91	56.17
Poor sleep quality	110	67.9

Comparison between study participants before and after the COVID era concerning anxiety, insomnia, and poor sleep was shown in Tables 5, 6, and 7. There was a significant change in the occurrence of anxiety in which among 137 participants without insomnia pre-COVID-19 pandemic, 82 patients had a recent occurrence of anxiety. Also, there was a significant change in the occurrence of insomnia in which among 143 participants without insomnia pre-COVID-19 pandemic, 65 patients had a recent occurrence of insomnia. Moreover, there was a significant change in the occurrence of poor sleep in which among 133 participants without poor sleep pre-COVID-19 pandemic, 88 participants had a recent occurrence of poor sleep, with a highly statistically significant difference, *p* < 0.001.

**Table 5:** Comparison between participants before and after COVID-19 pandemic in relation to anxiety

			Anxiety during COVID-19		Total	*P*
			Absent	Present		
Anxiety pre-COVID-19	Absent	No	78	65	143	< 0.001
		% of total	48.1%	40.1%	88.3%	
	Present	No	4	15	19	
		% of total	2.5%	9.3%	11.7%	
Total		No	82	80	162	
		% of total	50.6%	49.4%	100.0%

**Table 6:** Comparison between study participants before and after COVID-19 pandemic in relation to insomnia

			Insomnia during COVID-19		Total	*P*
			Absent	Present		
Insomnia pre-COVID-19	Absent	No	55	82	137	< 0.001
		% of total	34.0%	50.6%	84.6%	
	Present	No	16	9	25	
		% of total	9.9%	5.6%	15.4%	
Total		No	71	91	162	
		% of total	43.8%	56.2%	100.0%

**Table 7:** Comparison between study participants before and after COVID-19 pandemic in relation to poor sleep

			Poor sleep post/during COVID-19		Total	*P*
			Absent	Present		
Poor sleep pre-COVID-19	Absent	Count	45	88	133	< 0.001
		% of total	27.8%	54.3%	82.1%	
	Present	Count	7	22	29	
		% of total	4.3%	13.6%	17.9%	
Total		Count	52	110	162	
		% of total	32.1%	67.9%	100.0%	

Among 106 participants without prior condition before the COVID-19 pandemic, 43.4% developed anxiety, 58.5% developed insomnia, and 65.1% developed poor sleep. Moreover, the cumulative effect of the COVID-19 pandemic on the study participants without prior condition revealed that one condition was recently developed after the pandemic: insomnia in 6.6%, anxiety in 5.7%, and poor sleep in 16%. Two conditions were developed after the pandemic: insomnia and poor sleep in 21.7%, anxiety and poor sleep in 7.5%, and insomnia and anxiety in 10.4%. The three conditions were de novo experienced in 19.8%, while 12.3% still are not influenced by the pandemic (Table 8).

**Table 8:** Impact of COVID-19 on 106 participants without prior affection before the current pandemic

		Frequency	%
Anxiety related to COVID-19	Absent	60	56.6
	Present	46	43.4
Insomnia related to COVID-19	Absent	44	41.5
	Present	62	58.5
Poor sleep related to COVID-19	Absent	37	34.9
	Present	69	65.1
**Impact of COVID-19**			
Poor sleep		17	16.0
Insomnia		7	6.6
Anxiety		6	5.7
Poor sleep and insomnia		23	21.7
Poor sleep and anxiety		8	7.5
Insomnia and anxiety		11	10.4
Poor sleep and insomnia and anxiety		21	19.8
No condition		13	12.3

**Fig. 1:**
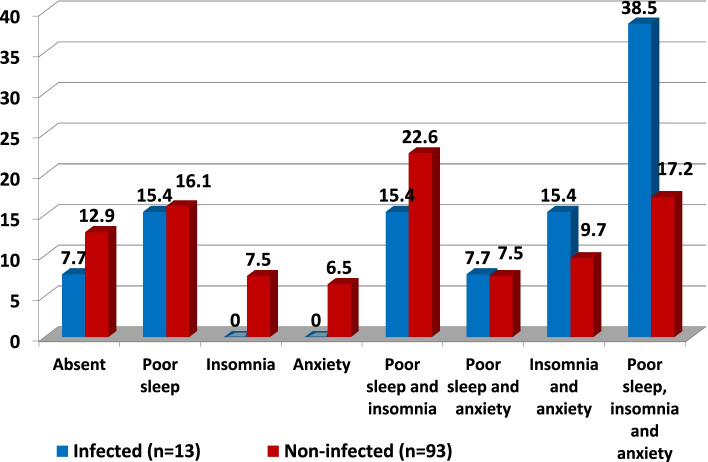
Impact of being got infected on de novo development of the combined mental health affection

**Fig. 2:**
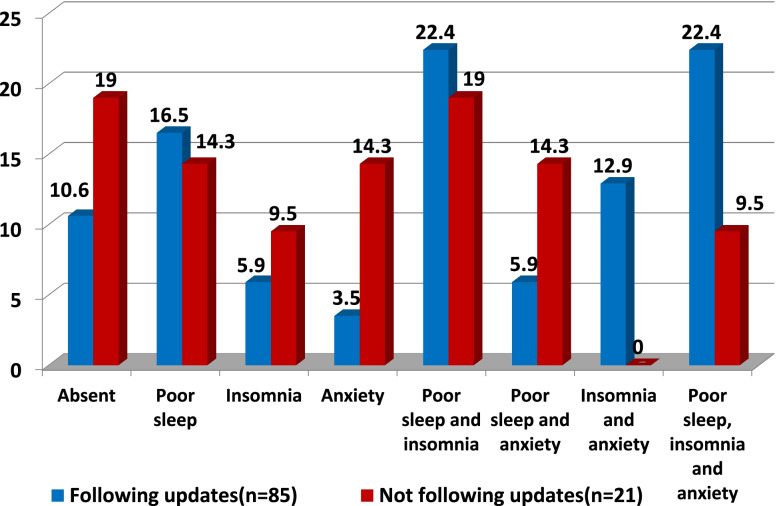
Impact of following daily COVID-19 updates and news on de novo development of the combined mental health affection

**Fig. 3:**
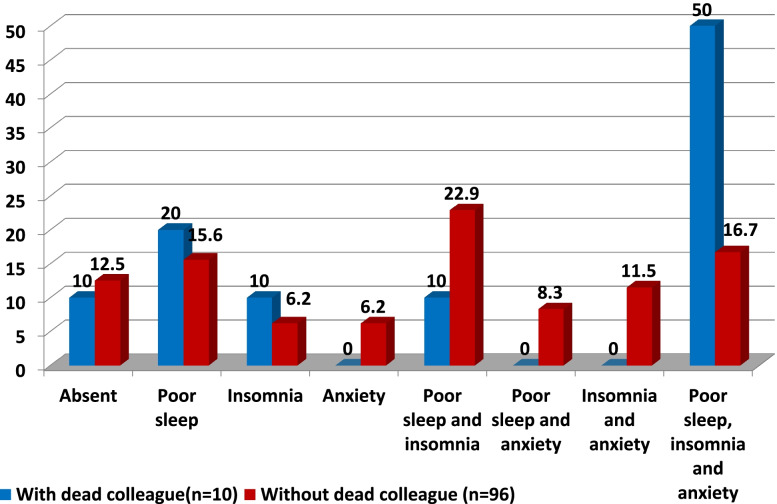
Impact of having colleague/friend died with COVID-19 on de novo development of the combined mental health affection

The impact of COVID-19-related factors on second-line HCWs was highlighted in Figs. 1, 2, and 3. Among 106 participants without prior condition, 22.4% of those who followed updates about COVID-19 on a daily basis developed de novo combined anxiety, insomnia, and poor sleep compared to only 9.5% of those who were not following updates. A total of 38.5% of the participants that had been infected with COVID-19 developed de novo combined anxiety, insomnia, and poor sleep compared to only 17.2% of those who had not been infected. A total of 50% of the participants who had a colleague/friend who died with COVID-19 developed de novo combined anxiety, insomnia, and poor sleep compared to only 16.7% of those who had not.

Correlation between sociodemographic and work-related characteristics with various mental health issues was shown in Table 9 and revealed an association between female sex with development of anxiety (*p* = 0.001). Moreover, being married to a HCW was linked with anxiety and insomnia (*p* < 0.001, 0.04, respectively). Also, there was a trend of increasing frequency of anxiety, insomnia, and poor sleep with those whose experience was ≤ 5 years (*p* < 0.001, 0.01, 0.04, respectively), while age and unchanged working hours were not associated with any mental health problems.

**Table 9:** Correlation between sociodemographic and work-related characteristics with various mental health issues in patients without prior affection

Variable		Anxiety (*n* = 46)	No anxiety (*n* = 60)	*P*	Insomnia (*n* = 62)	No insomnia (*n* = 44)	*P*	Poor sleep (*n* = 69)	No poor sleep (*n* = 37)	*P*
Age (years)		33.78 ± 5.7	34.92 ± 3.28	0.65	35.1 ± 2.69	33.92 ± 6.83	0.71	33.87 ± 4.15	34.69 ± 3.79	0.59
Sex (female)		36 (64.3%)	20 (35.7%)	0.001	33 (58.9%)	23 (41.1%)	0.92	38 (67.9%)	18 (32.1%)	0.53
Partner HCW		30 (71.4%)	12 (28.6%)	< .001	27 (64.3%)	15 (35.7%)	0.04	31 (73.8%)	11 (26.2%)	0.13
Experience (years)	≤ 5	38 (68%)	18 (32%)	< .001	40 (75%)	15 (25%)	0.01	40 (75.5%)	13 (24.5%)	0.04
	6–10	3 (16.7%)	15 (83.3%)		9 (90%)	9 (10%)		8 (50%)	8 (50%)	
	> 10	5 (15.6%)	27 (84.4%)		13 (66.7%)	20 (33.3%)		21 (56.8%)	16 (43.2%)	
Wok hours/week	≤ 48	39 (43%)	52 (57%)	0.78	53 (59%)	37 (41%)	0.48	57 (64.8)	31 (35.2)	0.88
	> 48	7 (46.6)	8 (53.3)		9 (56.2%)	7 (43.8%)		12 (66.7)	6 (33.3)	

## Discussion

Human behavior worldwide changed after the lockdown measures in response to the COVID-19 pandemic [1]. Believably, environmental and social changes introduced by the pandemic were shown to affect sleep timing and duration in recent studies among different populations and have resulted in an unprecedented psychological impact on healthcare workers, who were already working under stressful conditions [2, 8–11]. Growing reports highlighted the impact of the COVID-19 pandemic on sleep quality, anxiety, insomnia, and other neuropsychiatric influences among the frontline healthcare workers as being on fire, facing the new enemy, and exposed to infection and severe complications up to death. This is a rich research point as the relationship looks bidirectional; improvement in mental health and sleep efficiency may reduce the negative impact of the current pandemic and vice versa [12–14].

On the other hand, to the best of our knowledge, no reports focusing on the second-line HCWs who are not in the direct battle with the new enemy and the implications of the pandemic on their mental health, quality of life with a suspected negative impact on their work performance, are still not studied efficiently. So, we conducted the current work to assess anxiety, insomnia, and poor sleep among second-line HCWs during the COVID-19 pandemic.

The main results in the current work are as follows: although being a second-line HCWs, there was a high prevalence of anxiety, insomnia, and poor sleep, during the COVID-19 era. There was a significant change in anxiety, insomnia, and poor sleep in participants after the current pandemic. Also, among participants without prior condition before the COVID-19 pandemic, 43.4% developed anxiety, 58.5% developed insomnia, and 65.1% developed poor sleep.

Moreover, the cumulative effect of the COVID-19 pandemic on the study participants without prior condition revealed that three conditions (anxiety, insomnia, & poor sleep) were de novo experienced in 19.8%, while 12.3% still are not affected by the pandemic. Among 106 participants without prior condition, participants that had been following daily pandemic updates, being infected with, or having a friend/colleague who died with COVID-19 developed higher frequencies of de novo combined anxiety, insomnia, and poor sleep compared to those who do not.

A wide variety of studies worldwide focused on frontline HCWs and the effect of the current pandemic on mental health issues. In a recent Jordanian study, approximately 1/3 of the studied frontline HCWs who engaged in direct management of COVID-19 patients reported severe symptoms of anxiety (29.5%), depression (34.5%), and insomnia (31.9%) [15].

Mental health outcomes among frontline and second-line HCWs during the COVID-19 pandemic were studied in Italy by Rossi and colleagues in 2020 [16]. And reported 49.38% of the respondents endorsed post-traumatic stress, 24.73% reported symptoms of depression, 19.80% had symptoms of anxiety, and 8.27% had insomnia.

In another study from Kuwait, the reported prevalence of poor sleep during the current pandemic was 78.8 [14].

In China, a considerable percent of participants reported symptoms of anxiety (560 [44.6%]) and insomnia (427 [34.0%])). Median [IQR] Insomnia Severity Index scores among frontline vs second-line workers were 6.0 [2.0–11.0] vs 4.0 [1.0–8.0]; *P* < 0.001. Multivariate regression analysis showed that frontline HCWs engaged in close contact and management care of COVID-19 patients were associated with an increased risk of anxiety (*OR*, 1.57; 95% *CI*, 1.22–2.02; *P* < 0.001) and insomnia (*OR*, 2.97; 95% *CI*, 1.92–4.60; *P* < 0.001) [17].

There was an association between female sex with development of anxiety. Moreover, being married to a HCW was linked with anxiety and insomnia. Also, there was a trend of increasing frequency of anxiety, insomnia, and poor sleep with those whose experience was ≤ 5 years, while age and unchanged working hours were not associated with any mental health problems.

Oteir et al. in their study reported a mean age of participants was 32.1 (± 5.8) years, and the majority were males (80.3%), with no observed differences based on gender, job title, marital status, or educational level. Moreover, in the multivariate linear regression, none of the independent factors was associated with various neuropsychiatric scores, and the only exception was increased severity of insomnia among paramedics [15].

In a Chinese cross-sectional study of 1257 HCWs from 34 hospitals concerning with COVID-19 management, a considerable association was reported between depression, anxiety, and insomnia in frontline female nurses who particularly worked in Wuhan [17].

Younger age and female sex were associated with all investigated outcomes except insomnia (e.g., anxiety for standardized age: odds ratio [OR], 0.60; 95% *CI*, 0.44–0.82; *P* = 0.001; perceived stress for standardized age: *OR*, 0.63; 95% *CI*, 0.46–0.85; *P* = 0.002; post-traumatic stress among women: *OR*, 2.31; 95% *CI*, 1.76–3.05; *P* < 0.001; depression among women: *OR*, 2.03; 95% *CI*, 1.44–2.87; *P* < 0.001). Being a frontline HCW was associated with PTSS (*OR*, 1.37; 95% *CI*, 1.05–1.80; *P* = 0.03) [16].

Among 106 participants without prior condition, 22.4% of those who followed updates about COVID-19 on a daily basis developed de novo combined anxiety, insomnia, and poor sleep compared to only 9.5% of those who were not following updates. A total of 38.5% of the participants that had been infected with COVID-19 developed de novo combined anxiety, insomnia, and poor sleep compared to only 17.2% of those who had not been infected. A total of 50% of the participants who had a colleague/friend who died with COVID-19 developed de novo combined anxiety, insomnia, and poor sleep compared to only 16.7% of those who had not.

In an Italian study, nurses and healthcare assistants were more likely to endorse severe insomnia (nurses: *OR*, 2.03; 95% *CI*, 1.14–3.59; *P* = 0.02; healthcare assistants: *OR*, 2.34; 95% *CI*, 1.06–5.18; *P* = 0.04). Having a colleague who died was associated with post-traumatic stress (*OR*, 2.60; 95% *CI*, 1.30–5.19; *P* = 0.007) and symptoms of depression (*OR*, 2.07; 95% *CI*, 1.05–4.07; *P* = 0.04) and insomnia (*OR*, 2.94; 95% *CI*, 1.21–7.18; *P* = 0.02); having a colleague hospitalized was associated with PTSS (*OR*, 1.54; 95% *CI*, 1.10–2.16; *P* = 0.01) and higher perceived stress (*OR*, 1.93; 95% *CI*, 1.30–2.85; *P* = 0.001); and having a colleague in quarantine was associated with PTSS (*OR*, 1.59; 95% *CI*, 1.21–2.09; *P* = 0.001), symptoms of depression (*OR*, 1.38; 95% *CI*, 1.00–1.90; *P* = 0.047), and higher perceived stress (*OR*, 1.66; 95% *CI*, 1.19–2.32; *P* = 0.002). Being exposed to contagion was associated with symptoms of depression (*OR*, 1.54; 95% *CI*, 1.11–2.14; *P* = 0.01 [16].

Frontline HCWs are the leading group affected by different mental health issues than before the current pandemic because of the higher chance of getting the infection with the unavailability of adequate personal protective equipment, high-stress level, and heavy daily workloads [14].

There had been strict lockdown measures to prevent the spread of the disease as guided by the WHO. As COVID-19 is highly contagious, social distancing and self-isolation were the best preventive measures to minimize the risk of spreading the infection. However, these precautionary strategies have a deleterious impact on emotional and mental health, such as the development of chronic insomnia. It can vary from simple mood changes, depression, and minor anxiety to very severe forms of mental issues such as severe depression and even suicide [18–20].

The home isolation itself is a psychological burden, and even the individual does not have clinical symptoms and remains physically well, they often suffer from adverse psychological effects [21].

Not only the frontline HCWs are at increased risk of sleep disturbances and mental health affection. In addition to the factors mentioned above, the second-line HCWS have other unique risks: first, the preexisting sleep and mental health issues before the pandemic due to heavy work schedules and frequent night shifts. This fragility makes them more susceptible to the deterioration or addition of a new health issue. Second, the second-line HCWs have a very likely chance to move forward to be a frontline one as a part of rotatory strategies or the need for more fighters in times of the pandemic peaks. Third, they are more interested in daily updates of disease and death rates, developing and failing many promising therapeutics supposed to achieve successful treatment.

To the best of our knowledge, this is the first study focusing on the second-line HCWs and the effects of the new pandemic on their mental health that can negatively affect health system quality. This can be a nucleus for further studies and planning for future therapeutic strategies.

This study’s limitations include the cross-sectional design; therefore, mental health affection was not evaluated during different time intervals. Moreover, it could be affected by the peak of COVID-19 cases. Finally, other variables that could affect mental health, e.g., depression and burnout, were not assessed. 

## Conclusion

Although being second-line HCWs, there was a significant change in anxiety, insomnia, and poor sleep in participants after the current pandemic. These findings warranted further monitoring and specific interventions to prevent long-term mental health-related consequences throughout the current pandemic.

## Data Availability

The datasets used and/or analyzed during the current study are available from the corresponding author on reasonable request.

## Abbreviations

HCWs: Healthcare workers
COVID-19: Coronavirus disease
SARS-CoV-2: Severe acute respiratory syndrome coronavirus 2
CI: Confidence interval
